# Supplementary material for: Field evaluation of rapid diagnostic tests to determine dengue serostatus in Timor-Leste
Source: PLoS Negl Trop Dis. 2022 Nov 7;16(11):e0010877. doi: 10.1371/journal.pntd.0010877 (PMC9671414; doi:10.1371/journal.pntd.0010877)
Supplement: S1 Fig — (PDF) [file pntd.0010877.s002.pdf]

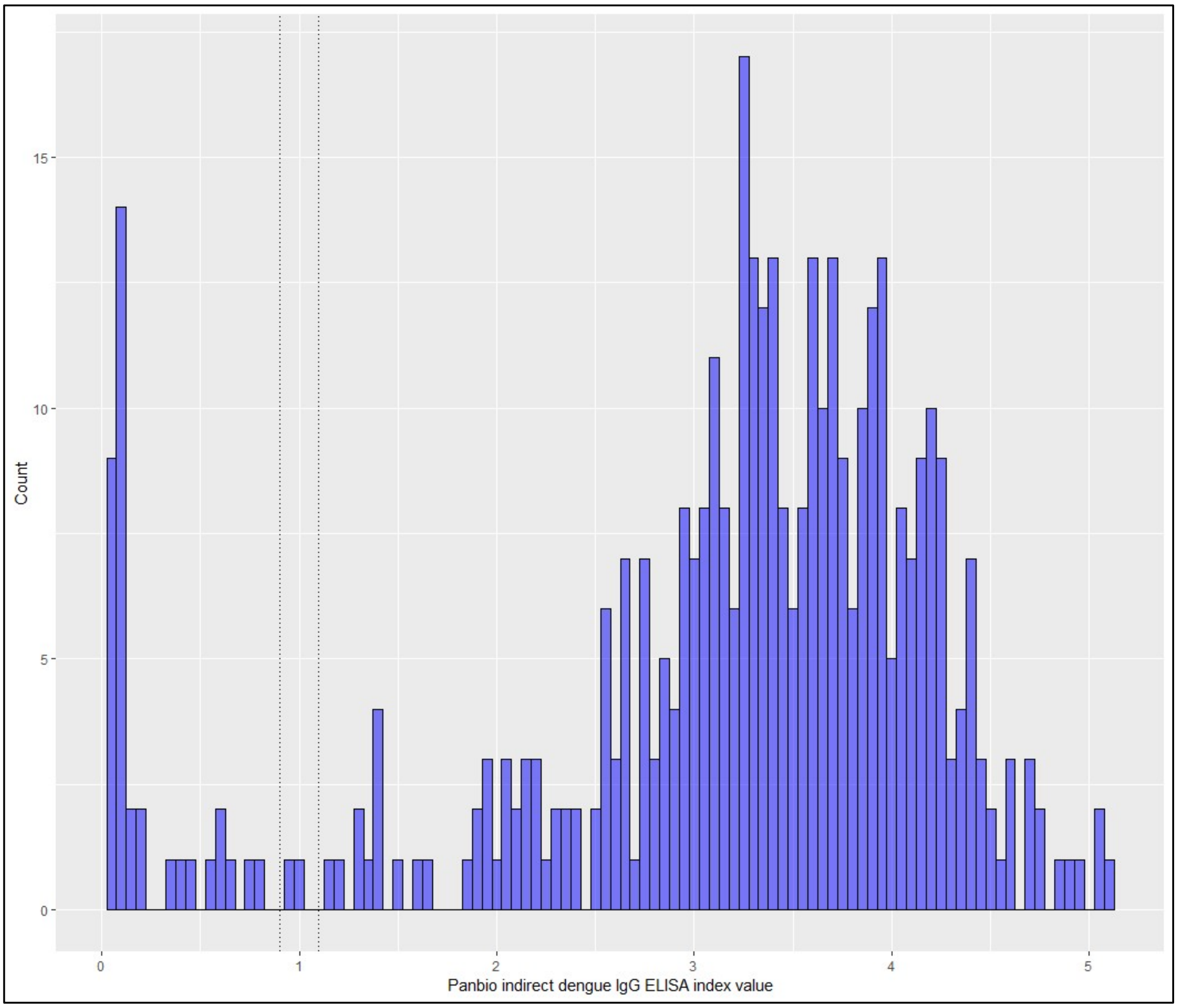

Appendix 1: Histogram showing antibody responses in serum samples from all individuals when tested using the Panbio™ indirect dengue IgG ELISA, which was used as the reference test in this study. The commercial cut-off values are shown by dotted lines. These were assessed visually to adequately distinguish two populations and were therefore adopted for the study: Samples with index value (IV) < 0.9 were assigned 'dengue IgG negative. Samples with IV > 1.1 were assigned dengue IgG positive. Samples with IV 0.9 – 1.1 were repeat-tested with the second result being used. If IV remained 0.9 – 1.1 on repeat testing, then the sample was assigned 'dengue IgG positive'
