# Supplementary material for: Field evaluation of rapid diagnostic tests to determine dengue serostatus in Timor-Leste
Source: PLoS Negl Trop Dis. 2022 Nov 7;16(11):e0010877. doi: 10.1371/journal.pntd.0010877 (PMC9671414; doi:10.1371/journal.pntd.0010877)
Supplement: Attachment — Submitted filename: Reviewer_comments_JB.docx [file pntd.0010877.s003.docx]

**Reviewer’s comments**

**Title: Field evaluation of rapid diagnostic tests to determine dengue serostatus in Timor-Leste**

**Overview:**

This is a well written and concise manuscript concerned with investigating the performance of dengue rapid tests, used in both field and laboratory conditions, against reference ELISA testing. Its purpose was to investigate whether such RDTs could be useful in field settings for pre-vaccination screening. Investigators found that RDTs had low sensitivity (yet high specificity) against ELISA testing for IgG which is an expected result (see major comments). The more interesting aspect of this research were the alterations to commercial RDT practice (extending assay times) to detect lower levels of IgG. This has more public health relevance as it reveals whether RDTs can be used to detect those with exposure to dengue (not just current infections) who represent good candidates for vaccination.

**Major comments**

My biggest concern is that you are evaluating RDTs against ELISAs that both have contrasting outcome interpretations and you have not made this clear from the onset. The Bioline/Biosensor RDTs evaluated in this study are concerned with diagnosing active (current) DENV infections. Thus they are limited to detect higher levels of IgM and IgG that occur only during active infections. In contrast, the reference Panbio indirect IgG ELISA are concerned with detecting circulating anti-DENV IgG which is indicative of a patient having a current or past (historical) DENV infection(s). Hence you are comparing tests that are diagnosing active infections to a reference test that diagnoses active or past infections which is not a meaningful comparison. As such we would fully expect the RDTs to have low sensitivity against such ELISAs as RDTs may just be capturing the proportion of the ELISA IgG+ population that are active infections (yet correctly missing those with just past exposure to DENV).

Therefore the more relevant aspect of this research is the modifications to RDT procedures (extending the time) to investigate whether they can be used to accurately detect those with active and past infections to dengue. I believe this should be the main aim of your study and not just determining how well RDTs capture IgG positivity against ELISAs. It would also be beneficial for the reader to state in the introduction how these contrasting tests are interpreted and what they should be used for.

Secondly, how sure are you the reference ELISA test for detecting IgG is accurate? The commercial cut off for IgG are (>1.1, 0.9-1.1, 0.9). Has the performance of this ELISA been assessed for their sensitivity/specificity for diagnosing dengue? Do these arbitrary cut offs truly represent seropositivity in your study setting? A histogram of antibody responses overlaid by the commercial cut offs would indicate whether your cut offs truly distinguish the seropositive/seronegative antibody populations. If they do not ,then I would suggest additional analysis is needed to obtain accurate ELISA thresholds indicative of antibody seropositivity including mixture modelling approaches [Ward 2022] [Bottomley 2021].

**Minor comments**

Abstract

- “Four-hundred-and-six participants (including 217 children) were recruited in the community.” Were these participants recruited at random?
- “Reference testing determined that 370 (91.1%) participants were seropositive” - seropositive to what? IgM and IgG? IgM or IgG?

Background

- “Several of these RDTs have since been evaluated for detecting prior dengue infection in healthy individuals, where concentrations of IgG are lower” – because IgG has waned over time? This would be clearer to the reader if you describe IgG antibody kinetics during and after a DENV infection before describing the RDTs limit of IgG detection.
- “Studies show high specificity, but RDTs generally have lower sensitivity than traditional serological techniques, and performance is highly variable between assays.” Are you referring to just IgG RDTs? Is this true for both IgG and IgM RDTs?
- “The primary aim of this study was to determine the sensitivity and specificity of two RDTs in determining dengue seropositivity from whole blood, when operated under field conditions, using serum enzyme-linked immunosorbent assay (ELISA) as the reference test.” I think it would be of benefit if you briefly describe what ‘field conditions’ mean. RDT administered by clinical/non-clinical health care workers? In clinical/non-clinical settings?

Methods

- What was the justification for sampling 200 adults and 200 children? Were there any sample size considerations?
- A brief description of dengue in the study setting, Difi, would be useful. What are the historical trends in dengue incidence? Is the environment suitable to support transmission? Are you expecting most individuals to have exposure to dengue?
- Sample collection – more details are required to better understand the field conditions under which these RDTs are being assessed. Did nurses collect blood during visits or were community members invited to give blood in a clinical setting? Were nurses provided with any training in RDT testing? Were RDT tests conducted in the presence of community members?
- “Serum samples were analysed using the Panbio indirect IgG ELISA at the NHL according to manufacturers’ instructions” – it is standard procedure to include a catalogue number here.
- “Participants were tested for the presence of dengue antibodies using two different commercially available dengue RDTs (Bioline™ Dengue IgG/IgM, Abbott and Standard™ Q Dengue IgM/IgG, SD Biosensor). These were chosen based their positive performance in previous studies (Bioline™)[10,11]” – If previously tested, it would be useful for the reader to include estimates of kit sensitivity/specificity.
- “If dengue IgG was detected by either assay (but IgM was not detected), participants were informed that they had likely been infected with dengue at some time in their lives, but not recently. If IgM (+/- IgG) was detected by either assay, participants were informed that there was evidence of current or recent dengue infection. These individuals were advised to seek medical attention if they experienced fever or other acute illness during the subsequent two weeks” – what is the justification for such interpretations? Is this according to commercial interpretations of kit results or your own?
- “To assess whether RDT insensitivity was likely to be due to an assay limit-of-detection (LOD) issue (as opposed to some other deficiency)” - What is an example of ‘some other deficiency’?

Results

- “Reference testing determined that 186/217 (85.7%) children and 184/189 (97.4%) adults were dengue seropositive (overall seropositivity = 91.1%).” – for clarity I would state this is IgG seropositivity.
- Table 1: Sensitivity, specificity, PPV and NPV values should be accompanied with 95%CIs. Without them it is difficult to decipher how reliable such estimates are.
- “Analysis of ELISA IVs as a proxy for antibody concentration” – it is unclear why this sub analysis was conducted. In the methods it states it was done to ‘To assess whether

RDT insensitivity was likely to be due to an assay limit-of-detection (LOD) issue (as opposed to some other deficiency)’. How does this analysis answer this question? If it does/does not answer this question it should be stated in the results section.

Discussion

- Table 3 – is it not correct to include results (albeit a review of literature table) in the discussion section. This should be included in the introduction/results section.
- “Previous studies have found that ELISAs which detect dengue IgG can also be insensitive, particularly in individuals who have lower antibody titres and/or where there is a monotypic antibody profile.[18] Importantly, the latter group of individuals are most likely to benefit most from vaccination but would be denied this if pre-vaccination screening was false negative.” – this has been investigated previously by [Biggs et al 2022]. After day 3 of disease, an IgG- RDT result, in the presence of a NS1/IgM+ RDT result, is highly indicative of primary infection.

**References**

- Biggs JR, Sy AK, Ashall J, et al. (2022) Combining rapid diagnostic tests to estimate primary and post-primary dengue immune status at the point of care. PLoS Negl Trop Dis 16(5): e0010365.
- Bottomley, C., Otiende, M., Uyoga, S. et al. (2021) Quantifying previous SARS-CoV-2 infection through mixture modelling of antibody levels. Nat Commun 12, 6196.
- Ward D, Gomes AR, Tetteh KKA, et al. (2022). Sero-epidemiological study of arbovirus infection following the 2015-2016 Zika virus outbreak in Cabo Verde. Sci Rep. 2022 Jul 9;12(1):11719. doi: 10.1038/s41598-022-16115-4. PMID: 35810191; PMCID: PMC9271056.
