# Supplementary material for: Field evaluation of rapid diagnostic tests to determine dengue serostatus in Timor-Leste
Source: PLoS Negl Trop Dis. 2022 Nov 7;16(11):e0010877. doi: 10.1371/journal.pntd.0010877 (PMC9671414; doi:10.1371/journal.pntd.0010877)
Supplement: Attachment — Submitted filename: Response to PLOS NTD reviewer comments.docx [file pntd.0010877.s004.docx]

**Dear Reviewers,**

**Thank you for your thorough and thoughtful review of our manuscript. Please find responses to each of your comments below (blue), and associated changes in the manuscript (now version 4).**

**Yours faithfully,**

**Dr Paul Arkell**

**Reviewer’s comments**

**Title: Field evaluation of rapid diagnostic tests to determine dengue serostatus in Timor-Leste**

**Overview:**

This is a well written and concise manuscript concerned with investigating the performance of dengue rapid tests, used in both field and laboratory conditions, against reference ELISA testing. Its purpose was to investigate whether such RDTs could be useful in field settings for pre-vaccination screening. Investigators found that RDTs had low sensitivity (yet high specificity) against ELISA testing for IgG which is an expected result (see major comments). The more interesting aspect of this research were the alterations to commercial RDT practice (extending assay times) to detect lower levels of IgG. This has more public health relevance as it reveals whether RDTs can be used to detect those with exposure to dengue (not just current infections) who represent good candidates for vaccination.

**Major comments**

My biggest concern is that you are evaluating RDTs against ELISAs that both have contrasting outcome interpretations and you have not made this clear from the onset. The Bioline/Biosensor RDTs evaluated in this study are concerned with diagnosing active (current) DENV infections. Thus they are limited to detect higher levels of IgM and IgG that occur only during active infections. In contrast, the reference Panbio indirect IgG ELISA are concerned with detecting circulating anti-DENV IgG which is indicative of a patient having a current or past (historical) DENV infection(s). Hence you are comparing tests that are diagnosing active infections to a reference test that diagnoses active or past infections which is not a meaningful comparison. As such we would fully expect the RDTs to have low sensitivity against such ELISAs as RDTs may just be capturing the proportion of the ELISA IgG+ population that are active infections (yet correctly missing those with just past exposure to DENV).

Therefore the more relevant aspect of this research is the modifications to RDT procedures (extending the time) to investigate whether they can be used to accurately detect those with active and past infections to dengue. I believe this should be the main aim of your study and not just determining how well RDTs capture IgG positivity against ELISAs. It would also be beneficial for the reader to state in the introduction how these contrasting tests are interpreted and what they should be used for.

Thank you for this comment. We agree that existing RDTs have been designed for diagnosis of dengue. The aim of this study was indeed to assess whether two commonly used RDTs could be repurposed for detecting previous infection in healthy individuals. An additional aim was to explore whether modification to RDT procedure could improve performance. We have therefore changed the passage in the introduction to read:

*“Existing RDTs which detect IgG have been designed for the diagnosis of dengue in patients with acute febrile illness and/or for differentiation of primary vs. secondary dengue. Performance is therefore likely to have been optimised based on high concentrations of IgG associated with acute infection. The potential for these devices to be repurposed for detecting previous dengue infection in healthy individuals (where typically IgG concentrations have waned over time and are lower) has been explored in previous studies. Devices have generally been shown to have high specificity but low sensitivity when compared to traditional serological techniques, with performance varying between assays.[10–14] …*

*…* *The primary aim of this study was to determine the sensitivity and specificity of two RDTs when repurposed to determine dengue seropositivity in healthy individuals. Secondary aims were to compare performance of devices when used under field conditions (i.e. whole blood tested by research nurses in the community, in the presence of participants) to performance when used under laboratory conditions (i.e. serum samples tested by laboratory scientists in a diagnostic laboratory setting), explore whether any deficiencies are likely to be in-part due to assay limits-of-detection, and explore whether performance could be enhanced by allowing assays to run beyond manufacturer recommendations (and read at a delayed time-point).*

We have also changed the abstract to read:

*Rapid diagnostic tests (RDTs) which can accurately determine individual dengue serostatus are needed for use in pre-vaccination screening. This study aimed to determine the performance of existing RDTs (which have been designed to detect levels of immunoglobulin G, IgG, associated with acute secondary dengue) when repurposed for detection of previous dengue infection (where concentrations of IgG are typically lower).*

Secondly, how sure are you the reference ELISA test for detecting IgG is accurate? The commercial cut off for IgG are (>1.1, 0.9-1.1, 0.9). Has the performance of this ELISA been assessed for their sensitivity/specificity for diagnosing dengue? Do these arbitrary cut offs truly represent seropositivity in your study setting? A histogram of antibody responses overlaid by the commercial cut offs would indicate whether your cut offs truly distinguish the seropositive/seronegative antibody populations. If they do not ,then I would suggest additional analysis is needed to obtain accurate ELISA thresholds indicative of antibody seropositivity including mixture modelling approaches [Ward 2022] [Bottomley 2021].

Thank you for this suggestion. As you rightly point out the reference test used (Panbio™ indirect IgG ELISA) has been designed for diagnosis of previous dengue infection. We are not aware that it has been validated for diagnosis of acute dengue. This is one of the reasons we chose this particular ELISA assay. Additionally, it has been used in in large seroprevalence studies previously, it has acceptable performance when compared to neutralization assays, and laboratory equipment and expertise for its were available at the NHL in Timor-Leste. We have added these details to the methods section:

*This assay was chosen because it has been* *has been designed for detection of previous dengue (as opposed to diagnosis of acute infection), it has been used in serosurveillance studies previously[15,16], it has acceptable performance when compared to virus neutralisation testing (considered most accurate in determining dengue serostatus and correlates best with outcomes after Dengvaxia®)[11,17], and because laboratory equipment and expertise for its use were available at the NHL.*

We also agree that using ELISA (and as-per instructions for use i.e. without performing any verification against another standard in the population being studied +/- adjustment of cutoff) is a limitation to our study. As suggested, we plotted a histogram of antibody responses with commercial cut-offs shown, which were assessed visually to adequately distinguish two populations. We added the following passages to the methods and discussion sections, respectively, and have included the histogram as an appendix:

*The appropriateness of these commercial serological cut-offs was assessed visually by plotting a histogram showing ELISA IVs from all serum samples in the study, which was assessed visually. The cut-off values appeared to adequately distinguish two populations of antibody responses and were therefore adopted into the study.*

*… while the chosen reference test (Panbio™ indirect IgG ELISA) has been designed for detection of previous dengue (as opposed to diagnosis of acute infection), and has been used in serosurveillance studies previously[15,16], it may have mis-assigned some samples. This could have occurred because the commercial serological cut-off was used rather than any* *population-specific alternative derived from the observed antibody responses. Furthermore, virus neutralisation assays may have been more accurate in determining dengue serostatus (and could correlate better with outcomes after Dengvaxia®) than ELISAs [11,17], however they were not available during this study which took place in Timor-Leste.*

**Minor comments**

Abstract

- “Four-hundred-and-six participants (including 217 children) were recruited in the community.” Were these participants recruited at random?

As described in the methods section these were the first 200 adults and first 200 children recruited into a anothe, ongoing serosurvey. We have changed the abstract to read:

*A convenience sample of…*

- “Reference testing determined that 370 (91.1%) participants were seropositive” - seropositive to what? IgM and IgG? IgM or IgG?

Thank you, this has been changed to read:

*…were dengue IgG seropositive*

Background

- “Several of these RDTs have since been evaluated for detecting prior dengue infection in healthy individuals, where concentrations of IgG are lower” – because IgG has waned over time? This would be clearer to the reader if you describe IgG antibody kinetics during and after a DENV infection before describing the RDTs limit of IgG detection.

Thank you. We amended this section to make the distinction clearer but have refrained from describing the humeral immune response to dengue infection in detail to try and keep our manuscript concise and on-topic. However, we would reconsider this if it is felt absolutely necessary:

*The potential for these devices to be repurposed for detecting previous dengue infection in healthy individuals (where typically IgG concentrations have waned over time and are lower) has been explored in previous studies.*

- “Studies show high specificity, but RDTs generally have lower sensitivity than traditional serological techniques, and performance is highly variable between assays.” Are you referring to just IgG RDTs? Is this true for both IgG and IgM RDTs?

Our literature review identified 7 studies which assessed RDTs for this purpose. These are summarised in Table 3. All studies only assessed devices based on their IgG determination (and when commercially available devices included IgM determination this was not included). We have included this information in Table 3 and also changed the background accordingly.

- “The primary aim of this study was to determine the sensitivity and specificity of two RDTs in determining dengue seropositivity from whole blood, when operated under field conditions, using serum enzyme-linked immunosorbent assay (ELISA) as the reference test.” I think it would be of benefit if you briefly describe what ‘field conditions’ mean. RDT administered by clinical/non-clinical health care workers? In clinical/non-clinical settings?

Thank you. We have changed the aims section which now includes the following:

*Secondary aims were to compare performance of devices when used under field conditions (i.e. whole blood tested by research nurses in the community, in the presence of participants) to performance when used under laboratory conditions (i.e. serum samples tested by laboratory scientists in a diagnostic laboratory setting)…*

Methods

- What was the justification for sampling 200 adults and 200 children? Were there any sample size considerations?

Thank you we have included sample size considerations in the methods section as follows:

*This sample size was considered adequate to estimate 70% sensitivity and 95% specificity of RDTs with 5% precision (alpha=0.05)[15]. Since there has been no previous population serological surveillance of dengue in Dili, seroprevalence was assumed to be 80% (i.e. similar to other urban Southeast Asian settings with high burden of dengue).[16]*

- A brief description of dengue in the study setting, Difi, would be useful. What are the historical trends in dengue incidence? Is the environment suitable to support transmission? Are you expecting most individuals to have exposure to dengue?

Please see the response above and the following passage which is in the introduction:

*Timor-Leste is an island nation in Southeast Asia where dengue causes seasonal outbreaks across all 13 municipalities and is a major cause of morbidity and mortality among children.[5]*

- Sample collection – more details are required to better understand the field conditions under which these RDTs are being assessed. Did nurses collect blood during visits or were community members invited to give blood in a clinical setting? Were nurses provided with any training in RDT testing? Were RDT tests conducted in the presence of community members?

We have included the following passage in the Participants and Testing with RDTs sections, respectively.

*Potential participants were visited at their homes.*

*Research nurses and laboratory scientists attended classroom teaching sessions on the safe and appropriate use the RDTs which included practicing operating each device and being observed, which was delivered by PA.*

- “Serum samples were analysed using the Panbio indirect IgG ELISA at the NHL according to manufacturers’ instructions” – it is standard procedure to include a catalogue number here.

Thank you we have included product code 01PE30

- “Participants were tested for the presence of dengue antibodies using two different commercially available dengue RDTs (Bioline™ Dengue IgG/IgM, Abbott and Standard™ Q Dengue IgM/IgG, SD Biosensor). These were chosen based their positive performance in previous studies (Bioline™)[10,11]” – If previously tested, it would be useful for the reader to include estimates of kit sensitivity/specificity.

We have included the range of sensitivities and specificities from previous studies in the text (see also table 3)

- “If dengue IgG was detected by either assay (but IgM was not detected), participants were informed that they had likely been infected with dengue at some time in their lives, but not recently. If IgM (+/- IgG) was detected by either assay, participants were informed that there was evidence of current or recent dengue infection. These individuals were advised to seek medical attention if they experienced fever or other acute illness during the subsequent two weeks” – what is the justification for such interpretations? Is this according to commercial interpretations of kit results or your own?

The following passage has been added:

*These interpretations were agreed by members of the research team who have backgrounds in medical microbiology, adult, and paediatric infectious disease (PA, JY and JF). They are consistent with interpretations suggested by the RDT instructions for use, with the exception of isolated IgG seropositivity which was considered significantly more likely to represent previous infection than acute dengue in this group of asymptomatic participants.*

- “To assess whether RDT insensitivity was likely to be due to an assay limit-of-detection (LOD) issue (as opposed to some other deficiency)” - What is an example of ‘some other deficiency’?

Thank you. Other potential additional problems relate to the sample (e.g. inhibition), assay (e.g. antibody epitope /antigen binding problems), or output (e.g. misinterpretation). However these may be co-occuring so we have removed the bracketed section.

Results

- “Reference testing determined that 186/217 (85.7%) children and 184/189 (97.4%) adults were dengue seropositive (overall seropositivity = 91.1%).” – for clarity I would state this is IgG seropositivity.

Thank you, changed

- Table 1: Sensitivity, specificity, PPV and NPV values should be accompanied with 95%CIs. Without them it is difficult to decipher how reliable such estimates are.

These have now been added.

- “Analysis of ELISA IVs as a proxy for antibody concentration” – it is unclear why this sub analysis was conducted. In the methods it states it was done to ‘To assess whether

RDT insensitivity was likely to be due to an assay limit-of-detection (LOD) issue (as opposed to some other deficiency)’. How does this analysis answer this question? If it does/does not answer this question it should be stated in the results section.

Thank you, the following has been added to the results section:

*This indicates the RDTs were more likely to accurately identify positive samples which have higher dengue IgG concentrations and hence RDT insensitivity is at least partially due to an assay limit-of-detection problem.*

Discussion

- Table 3 – is it not correct to include results (albeit a review of literature table) in the discussion section. This should be included in the introduction/results section.

Thank you this has been moved to discussion

- “Previous studies have found that ELISAs which detect dengue IgG can also be insensitive, particularly in individuals who have lower antibody titres and/or where there is a monotypic antibody profile.[18] Importantly, the latter group of individuals are most likely to benefit most from vaccination but would be denied this if pre-vaccination screening was false negative.” – this has been investigated previously by [Biggs et al 2022]. After day 3 of disease, an IgG- RDT result, in the presence of a NS1/IgM+ RDT result, is highly indicative of primary infection.

Thank you for this comment. The paper cited is concerned with the use of RDTs to determine primary and post-primary immune status in individuals who have suspected acute dengue infection, rather than healthy individuals who may be tested months or years after their infection. In the former situation IgG RDTs may be negative in primary infection because tests are done before the IgG response has occurred (less likely in post-primary infection because of the quick anamnestic IgG response). In the latter situation IgG RDTs are more likely to be (falsely) negative because the IgG response has waned (an effect thought to happen less quickly in post-primary infection (i.e. after humeral immune system has had two stimuli). The reference has been added.

**References**

- Biggs JR, Sy AK, Ashall J, et al. (2022) Combining rapid diagnostic tests to estimate primary and post-primary dengue immune status at the point of care. PLoS Negl Trop Dis 16(5): e0010365.
- Bottomley, C., Otiende, M., Uyoga, S. et al. (2021) Quantifying previous SARS-CoV-2 infection through mixture modelling of antibody levels. Nat Commun 12, 6196.
- Ward D, Gomes AR, Tetteh KKA, et al. (2022). Sero-epidemiological study of arbovirus infection following the 2015-2016 Zika virus outbreak in Cabo Verde. Sci Rep. 2022 Jul 9;12(1):11719. doi: 10.1038/s41598-022-16115-4. PMID: 35810191; PMCID: PMC9271056.

Reviewer's Responses to Questions

Reviewer #2: This manuscript describes a study to assess the performance of two rapid diagnostic tests (RDTs) to determine dengue serostatus for pre-vaccination screening under field and laboratory conditions in Timor-Leste. The RDTs were performed using whole blood collected from 406 participants, of whom 217 were children. The results were compared to serum samples from the same individuals and tested at the National Health Laboratory using an enzyme-linked immunosorbent assay (ELISA) on serum samples as the comparator. The authors found that the RDTs had low sensitivity which can be improved by using serum and by extending the window for reading the results.

This is a well written paper on a topic that is critically important for the deployment of the only approved dengue vaccine, that can save lives. The study objectives are clearly stated, the study was well designed and the methods appropriate for the study objectives.

There are no ethical or regulatory issues.

Reviewer #2: The results are clearly presented in tables that are easy to understand and interpret and figures that are good quality.

As the interpretation of RDT results are subjective, the authors should previde details on training of field staff to perform the tests and whether external quality assessment was carried out to ensure proficiency of staff in performing the tests and interpreting the test results.

Thank you for this comment. Research nurses and laboratory scientists involved in this study attended classroom training sessions. However, due to resource constraints there was no formal programme of quality assurance and this is a limitation of the study. These details have been added to the methods and discussion section respectively:

*Research nurses and laboratory scientists attended classroom teaching sessions on the safe and appropriate use the RDTs which included practicing operating each device and being observed, which was delivered by PA.*

*Field workers in the present study received classroom training in the use of both RDTs and were observed to ensure proper technique, but there was no formal programme of quality assurance to ensure correct and consistent visual interpretation.*

Reviewer #2: The conclusions are supported by the data but there are a few points that should be discussed in more detail:

1.	Reference standard: As there is no reference materials for determining dengue seropositivity, the choice of the assay as the comparator clearly affects the test performance. As shown in Table 3, previous studies use different reference standards, the authors should discuss the different reference standards used in previous studies and how they may affect the study results.

Thank you for this comment. We agree that setting a reference standard is challenging for studies such as ours. We have updated the manuscript in several places to address your comment and others from reviewer 2.

The background now reads:

*Reference test: Serum samples were analysed using the Panbio™ indirect IgG ELISA (product code 01PE30). This assay was chosen because it has been has been designed for detection of previous dengue (as opposed to diagnosis of acute dengue infection), it has been used in serosurveillance studies previously[20,21], it has acceptable performance when compared to virus neutralisation testing (considered most accurate in determining dengue serostatus and correlates best with outcomes after Dengvaxia®)[11,22], and because laboratory equipment and expertise for its use were available at the NHL. Testing was performed in accordance with manufacturers’ instructions: A cut-off value was calculated for each run by multiplying the calibration factor (batch-specific) by the mean optical densities (ODs) of calibrator material (run in triplicate). An index value (IV) was then calculated by dividing the sample OD by the cut-off value. Samples with IV < 0.9 were assigned ‘dengue IgG negative’. Samples with index value > 1.1 were assigned dengue IgG positive. Samples with IV 0.9 – 1.1 were repeat-tested with the second result being used. If IV remained 0.9 – 1.1 on repeat testing then the sample was assigned ‘dengue IgG positive’. The appropriateness of these commercial serological cut-offs was assessed visually by plotting a histogram showing ELISA IVs from all serum samples in the study, which was assessed visually. The cut-off values appeared to adequately distinguish two populations of antibody responses and were therefore adopted into the study.*

The discussion now reads:

… while the chosen reference test (Panbio™ indirect IgG ELISA) has been designed for detection of previous dengue (as opposed to diagnosis of acute infection), and has been used in serosurveillance studies previously[20,21], it may have mis-assigned some samples. This could have occurred because the commercial serological cut-off was used rather than any population-specific alternative derived from the observed antibody responses. Furthermore, virus neutralisation assays may have been more accurate in determining dengue serostatus (and could correlate better with outcomes after Dengvaxia®) than ELISAs [11,22], however they were not available during this study which took place in Timor-Leste.

2.	Discussion: It is well known that IgG antibody responses to different flaviviruses are known to be cross-reactive. The authors should discuss the implications of false positive and false negative results from the use of the RDTs to determine dengue serostatus.

The following passage has been added to the discussion:

*Although this study found RDTs to have high specificity, false positive results were observed. These could have been due to antibodies against other flaviviruses which may be circulating in Timor-Leste including zika and Japanese encephalitis viruses, which commonly cross-react in serological assays.[24] If used in pre-vaccination screening, RDTs which give false positive results would give rise to inappropriate vaccine administration. As such, it would be important to further characterise any false positive samples from candidate assays and perform more extensive specificity testing using panels of samples which are known to have potentially cross-reacting antibodies.*

3.	The WHO Strategic Advisory Group of Experts (SAGE) on Immunization issued recommendations in April 2016 to introduce the dengue vaccine (Dengvaxia®) in geographical settings (national or subnational) with high endemicity only, as indicated by seroprevalence of >70% in the age group targeted for vaccination. The findings from this study population showed seropositivity rates in excess of 85%, the authors should discuss whether there is still a need for Timor Leste to conduct pre-screening for vaccine introduction.

Thank you. We have added the following passage to the discussion:

*Results from the ongoing population-representative serosurvey (from which this study took a convenience sample) will therefore be crucial in determining the most appropriate disease control strategy. If seroprevalence in the vaccine candidate age-group is very high (>70%), universal administration of Dengvaxia® without pre-vaccination screening may be appropriate, because the number of severe cases prevented in those who were seropositive would likely be substantially greater than the excess number that occurred in those who were seronegative.[7]*

Reviewer #2: See above

Reviewer #2: The evaluation of the performance of dengue RDTs to determine dengue serostatus is not new but the data on possible reasons for low sensitivity and how to improve it are useful.
